# The effect of octopaminergic compounds on the behaviour and transmission of *Gyrodactylus*

**DOI:** 10.1186/1756-3305-4-207

**Published:** 2011-10-27

**Authors:** Adam J Brooker, Mayra I Grano Maldonado, Stephen Irving, James E Bron, Matthew Longshaw, Andrew P Shinn

**Affiliations:** 1 Institute of Aquaculture, University of Stirling, Stirling FK9 4LA, UK; 2 Cefas Weymouth Laboratory, The Nothe, Barrack Road, Weymouth, DT4 8UB, UK

**Keywords:** *Gyrodactylus*, octopamine, behaviour, toxicology

## Abstract

**Background:**

The high transmission potential of species belonging to the monogenean parasite genus *Gyrodactylus*, coupled with their high fecundity, allows them to rapidly colonise new hosts and to increase in number. One gyrodactylid, *Gyrodactylus salaris *Malmberg, 1957, has been responsible for devastation of Altantic salmon (*Salmo salar *L.) populations in a number of Norwegian rivers. Current methods of eradicating *G. salaris *from river systems centre around the use of non-specific biocides, such as rotenone and aluminium sulphate.

Although transmission routes in gyrodactylids have been studied extensively, the behaviour of individual parasites has received little attention. Specimens of *Gyrodactylus gasterostei *Gläser, 1974 and *G. arcuatus *Bychowsky, 1933, were collected from the skin of their host, the three-spined stickleback (*Gasterosteus aculeatus *L.), and permitted to attach to the substrate. The movements of individual parasites were recorded and analysed.

**Results:**

The behaviour patterns of the two species were similar and parasites were more active in red light and darkness than in white light. Four octopaminergic compounds were tested and all four inhibited the movements of parasites. Treatment ultimately led to death at low concentrations (0.2 μM), although prolonged exposure was necessary in some instances.

**Conclusions:**

Octopaminergic compounds may affect the parasite's ability to locate and remain on its host and these or related compounds might provide alternative or supplementary treatments for the control of *G. salaris *infections. With more research there is potential for use of octopaminergic compounds, which have minimal effects on the host or its environment, as parasite-specific treatments against *G. salaris *infections.

## Background

As *Gyrodactylus *von Nordmann, 1832 (Monogenea) has no specific transmission stage in its life-cycle, movement between hosts must be achieved by strategies employed by the adult. Bakke *et al. *[1] suggested four routes by which gyrodactylids could transfer to a new host: (i) via contact with live hosts, (ii) via dead hosts, (iii) by detached parasites drifting in the water column, and (iv) by parasites attached to the substrate. This transmission potential, coupled with their high fecundity allows gyrodactylids to rapidly colonise new river systems [1,2]. Although transmission routes in gyrodactylids have been studied extensively, few workers have investigated the behaviour of individual gyrodactylids.

*Gyrodactylus salaris *Malmberg, 1957 has devastated Atlantic salmon (*Salmo salar *L.) populations where it is present in North European rivers [3] and currently the only method of eradicating *G. salaris *from river systems is by using biocides, such as rotenone. However, this is devastating for the river habitat and, once it has recovered, *G. salaris *can re-colonise the river if measures are not taken to prevent its re-introduction [2]. Consequently, the focus of research is moving towards finding alternative methods to control *G. salaris*, which target the pathogen without seriously affecting the river ecosystem. This requires an increased understanding of gyrodactylid biology and behaviour [4].

In the control of other pathogens, chemical treatments often target specific stages of the life-cycle, which can be exploited to reduce the survival or infectivity of the parasites *e.g*. teflubenzuron is used to disrupt the moult of sea lice (*Lepeophtheirus salmonis *Krøyer, 1837 and *Caligus elongatus *Nordmann, 1832) [5]. Neurotransmitter receptor agonists/antagonists are compounds that elicit a response by binding to a postsynaptic receptor (*e.g*. on muscle or nerve) and mimicking or blocking the natural transmitter. In this study, the effect of octopaminergic receptor agonists/antagonists on gyrodactylids was investigated. It is suggested that exposing gyrodactylids to these compounds may affect their ability to attach to a host, rendering them immobile and unable to infect a host.

In order to investigate the effect of octopaminergic chemicals on the behaviour of gyrodactylids, it was necessary to develop a bioassay to observe their behaviour. Therefore, the objectives of the study were to: 1) develop a system for recording and observing the movements of gyrodactylids under different lighting conditions; 2) determine optimum lighting conditions for observing the behaviour of gyrodactylids, by comparing their movements under white light, red light and in dark conditions; and 3) determine the efficacy of the four octopaminergic compounds on detached gyrodactylid behaviour.

## Materials and methods

### Source of parasites

As *Gyrodactylus salaris *is a notifiable pathogen in the UK, it was not possible to acquire them for use in this study and therefore gyrodactylids from three-spined sticklebacks (*Gasterosteus aculeatus *L.), which are easily obtainable, were used as a gyrodactylid model. Two species of *Gyrodactylus *were identified from sticklebacks, *G. gasterostei *Gläser, 1974 and *G. arcuatus *Bychowsky, 1933, although the former were in the majority (80% and 20%, respectively). Both species were used in the behaviour experiments.

Three-spined sticklebacks were netted from a tributary of the River Forth, Stirlingshire (56° 06' 37.77" N, 3° 58' 25.25" W) and maintained at 10°C in 30 litre, static tanks in an aquarium facility at the Institute of Aquaculture, University of Stirling. A 50% water change was carried out daily, using water collected from Loch Airthrey (56° 08' 39.53" N, 3° 53' 51.20" W) and the sticklebacks were fed *ad libitum *with frozen bloodworm (Gamma, Chorleywood, UK). *Gyrodactylus *for use in the behaviour experiments were removed from the sticklebacks using triangular mounted surgical needles (size 16, Barber of Sheffield, UK). Parasites were identified to species level using standard descriptions. Once the behaviour of each gyrodactylid had been determined, it was fixed and mounted in ammonium picrate glycerine according to the method detailed by Malmberg [6], identified and its maturity status determined (*i.e*. presence or absence of a male copulatory organ and/or an embryo *in utero*).

### Investigation of lighting conditions

Initially, a simple experiment was undertaken to determine the activity of gyrodactylids under light and dark conditions. A mark was made on the underside of a 9 cm diameter Petri dish using a permanent marker and a single *Gyrodactylus *was placed onto the mark in the Petri dish filled with stream water at 10°C. Parasites attached themselves by the haptor and twenty replicates of each were maintained in either ambient light (2800 lux) or dark conditions (0 lux). The replicates in ambient light were placed inside a cotton light-diffusing box to scatter the light and eliminate any directional cues. After three hours the straight line distance between the final position of the *Gyrodactylus *and the initial mark was measured.

### *Gyrodactylus *tracking

**Figure 1:**
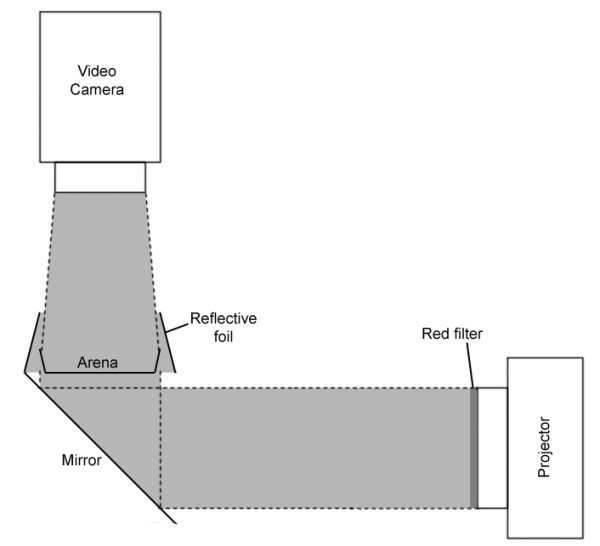
**Diagram of the experimental setup used to record the behaviour of detached specimens of *Gyrodactylus *under various lighting conditions or when exposed to a range of muscle agonists**.

An experimental system was constructed to record the behaviour of individual *Gyrodactylus *(Figure 1). This consisted of a 110 mm section of PVC pipe with a divider inserted inside the pipe. A circular hole 52 mm in diameter was cut in the divider and a mirror was placed underneath the divider at an angle of 45°. A 5 cm diameter Petri dish with a painted matt black base was placed onto supports surrounding the circular hole. Light was provided by a Carousel S 150W slide projector, which was directed onto the mirror, deflecting the light up through the divider and around the Petri dish. A foil cone set at an angle of ~30° directed light back into the centre of the Petri dish, forming a ring of incident light. This allowed the gyrodactylid to be detected in the arena and eliminated any directional light cue as the light level was consistent around the whole dish (Figure 1). A Canon MiniDV MD205 video camera was mounted on a stand above the arena to record the movements of the *Gyrodactylus*. Inflated circular rubber inner tubes measuring 20 and 50 cm in diameter were placed underneath the projector and the tray containing the light chamber to dampen vibrations from the projector.

For each replicate a new 5 cm diameter painted Petri dish was filled with 10 ml of 0.2 μm filtered stream water at 10°C and a single *Gyrodactylus *was placed into the centre of the arena using a Gilson pipette. It was then placed into the light chamber and left to settle for 20 minutes. The subsequent behaviour of the parasite was then recorded (T_20-50 _mins) onto MiniDV cassettes, using the video camera, for 30 minutes, before being fixed and mounted onto a glass slide. Ten replicates were recorded in white light (~2800 lux) and ten in red light, using a Hoya 600 nm (590-2750 nm) red photographic filter placed over the projector lens.

The 30 minute videos were converted to digital video files in.avi format using Windows Moviemaker software (version 2.1.4028.0, Microsoft Corporation, 2007). Individual frames in bitmap format were extracted using Bink and Smacker software (Bink version 1.9L, Smacker version 4.2d, RAD Game Tools Inc., 2009) at a frame rate of 1 frame per 5 seconds. Shade correction and segment analysis of the image set was performed in KS300 software (version 3.0 Carl Zeiss Vision GmbH, 1997) to facilitate the tracking of the parasite. Paratrack software (version 2.4, A. Brooker, University of Stirling, 2007) was used to track the movements of the parasite in each frame, creating an image of the gyrodactylid's movements and a text file containing a list of co-ordinates of the parasite's location in each frame. Once the parasites had been tracked the lists of co-ordinates were time averaged over three steps (15 seconds) to smooth the data, removing any bias in the calculated behaviour parameters as a result of exploratory extensions by the gyrodactylids whilst their haptors are stationary. The resultant co-ordinates were then used to calculate behavioural information including the mean and maximum velocity of each parasite, the distance travelled, turn rate, meander and heading. Fractal dimensions, which are a measure of track complexity, were also calculated for the parasite tracks using the 'box counting' method [7,8]. These operations were all undertaken using the Paratrack software. Principal Component Analysis (PCA -Statistica 6.1 software, 2004, Statsoft Inc., USA) was used to investigate differences between gyrodactylid movements in white and red light.

### Chemical efficacy

The following four octopaminergic compounds were tested in this trial: (±)-octopamine hydrochloride (C_8_H_11_NO_2_.ClH; O0250 Sigma), clonidine hydrochloride (C_9_H_9_Cl_2_N_3_.ClH; C7897 Sigma), amitraz (N-methylbis-(2,4-xylyl iminomethyl) amine, C_19_H_23_N_3_; 45323 Riedel-de Haën/Sigma) and chlordimeform (C_10_H_13_ClN_2_; 35913 Riedel-de Haën/Sigma). Chlordimeform was selected as a positive reference as it is known to be effective in the control of invertebrates and is toxic to aquatic life [9]. Octopamine is a biogenic monoamine found predominantly in invertebrates and modulates physiological activity by binding to adrenoceptors. In invertebrates it acts as a neurohormone, a neuromodulator or a neurotransmitter and modulates almost every physiological process [10]. In vertebrates noradrenaline is homologous to octopamine in invertebrates. Octopamine is found at concentrations less than 1% of noradrenaline in vertebrates, with its physiological activity being only 1-2% of noradrenaline [11]. Clonidine is a centrally-acting α-adrenergic receptor agonist and is known to reduce involuntary muscle contractions, or tics, in humans by binding to α2-adrenergic receptors [12]. Its mode of action is inhibition of adrenergic receptors, which results in reduced motor activity [13]. Amitraz and chlordimeform belong to a group of insecticides/acaricides (formamidines) whose mode of action is by interaction with octopamine receptors [13]. They work by mimicking the action of octopamine (centrally and at the neuromuscular junction) in invertebrates [14]. Amitraz acts as a receptor agonist, whereas chlordimeform has an antagonistic effect [15,13]. Although certain groups of invertebrates have been shown to be particularly sensitive to formamidine compounds (Acarines, Lepidoptera and Hemiptera), vertebrates in general are relatively insensitive [16]. Both chemicals have anthelmintic properties [17] and have been shown to induce hyperexcitation and detachment of feeding ticks [18,19].

As the efficacy of the four octopaminergic compounds on *Gyrodactylus *was unknown, a simple dose ranging exposure experiment was carried out using serial dilutions of each chemical with distilled water prepared in concentrations of 32, 16, 8, 4 and 2 μM plus a control consisting of distilled water only. One ml of each of these dilutions was pipetted into 5 cm diameter Petri dishes containing 9 ml of filtered stream water at 10°C to give final concentrations of 3.2, 1.6, 0.8, 0.4 and 0.2 μM. A single *Gyrodactylus *specimen was introduced into each Petri dish, which were then kept in an incubator at 10°C. Each chemical concentration was replicated 15 times. The parasites were checked after 24 and 48 h and recorded as alive, affected (*i.e*. not attached and showing muscular spasms), moribund (*i.e*. not attached, curled up and showing minute muscular contractions) or dead (*i.e*. no response to physical stimulus). After 48 h the gyrodactylids were preserved in ethanol for future identification and maturity assessment. After applying Abbot's correction factor [20] to account for control mortality, probit analysis (Minitab 13.1 Software, 2000, Minitab Inc., USA) was used to calculate 24 h and 48 h 50% effective concentration (EC50) values for each of the octopaminergic compounds. Where EC50 values are given, figures in parentheses are fiducial limits.

## Results

### Lighting conditions

**Figure 2:**
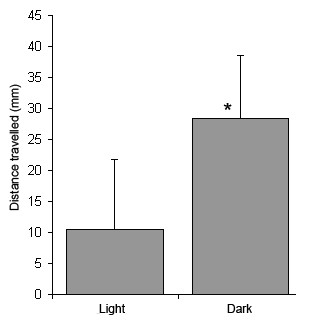
**Distance travelled by *Gyrodactylus *after 3 h in light (n = 19) and dark (n = 19) conditions**. Bars = 1 S.D., * = significant difference from white light response (p < 0.001).

As there was no significant difference between the distances travelled by each species of *Gyrodactylus *the data were combined. The investigation suggested that *Gyrodactylus *are more active in dark than in light conditions (P = < 0.001, one-way ANOVA) (Figure 2). After three hours, parasites in dark conditions moved a mean distance of 28.37 ± 10.18 mm from their starting point, whereas those in white light conditions moved only 11.8 ± 10.13 mm.

### Tracking

**Figure 3:**
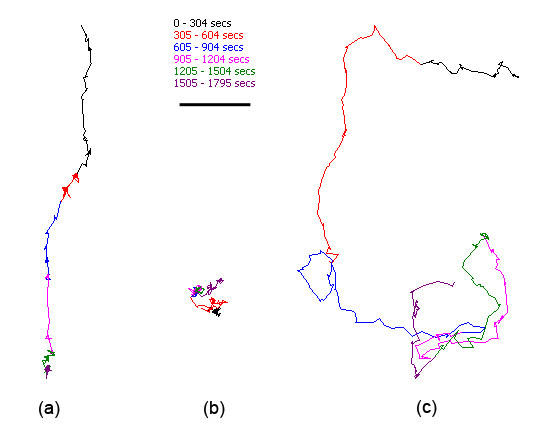
**Thirty minute *Gyrodactylus *tracks, showing different types of behaviour**. (a) Linear movement, (b) limited movement and (c) long sinuous movements with track crossovers. Bar = 5 mm.

Observation of the 30 minute tracks of individual *Gyrodactylus *showed several different behaviour patterns that were common to both species of *Gyrodactylus *tested: The most common behaviour involved moving in one direction with little deviation from the chosen heading (Figure 3a); the movements of some individuals were confined to a very small area around the starting point (Figure 3b); the final behaviour pattern can be described as extensive sinuous movements, with several path crossovers (Figure 3c). Individuals recorded in white light conditions appeared to display the first and second behaviour patterns, whereas individuals recorded under red light appeared to have longer, more sinuous tracks. No correlation was found between the different behaviour types and the species or maturity status of the individual gyrodactylids.

**Figure 4:**
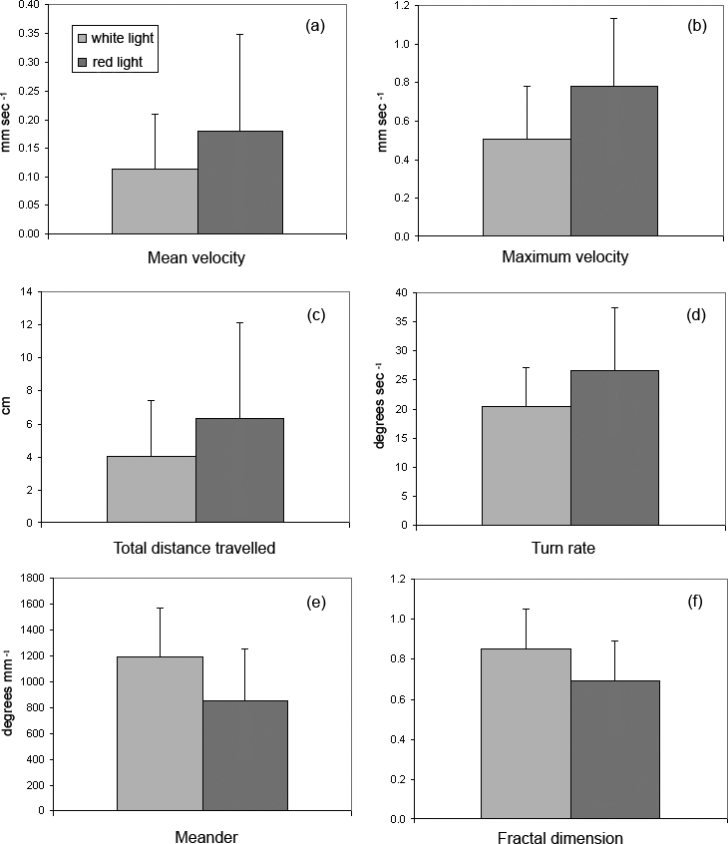
**Behaviour parameters for *Gyrodactylus *recorded in white and red light conditions (n = 10)**. (a) mean velocity; (b) maximum velocity; (c) distance travelled; (c) turn rate; (e) meander and (f) fractal dimension. Bars = 1 S.D.

Analysis of the tracks revealed that gyrodactylids in red light (n = 10) had a higher mean velocity (0.18 ± 0.17 mm/sec) and maximum velocity (0.78 ± 0.35 mm/sec), travelled further (6.32 ± 5.81 cm) and had a higher turn rate (± 26.6 degrees/sec) compared to those in white light (n = 10), which had a mean velocity of 0.11 ± 0.10 mm/sec, maximum velocity of 0.51 ± 0.28 mm/sec, travelling distance of 4.04 ± 3.35 cm and turn rate of 20.35 ± 6.79 degrees/sec (Figure 4). However, none of these values were significantly different (one-way ANOVA). Fractal dimensions and meander were lower for gyrodactylids in red light (0.69 ± 0.2 and 856 ± 397 degrees/mm) than for those in white light (0.85 ± 0.2 and 1195 ± 373 degrees/mm), indicating less complex tracks for those in red light, although again none of these values were significantly different (one-way ANOVA). The behavioural data was analysed (one-way ANOVA) for differences according to species of *Gyrodactylus *and maturity status, but none were found and no patterns in the data were apparent, suggesting that the different behaviours observed in this study are unrelated to the species and/or age of the gyrodactylids.

**Figure 5:**
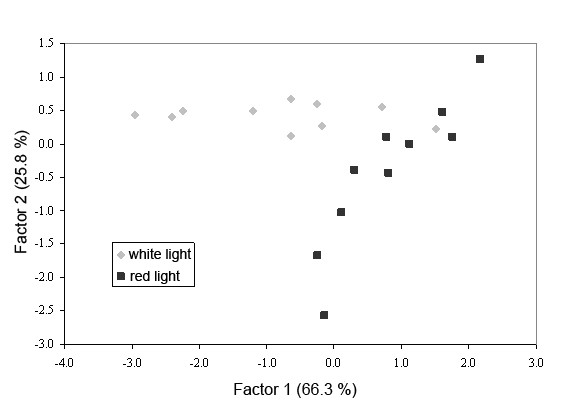
**Principal Component Analysis of maximum velocity, meander and fractal dimension for gyrodactylids exposed to white light (n = 10) and red light (n = 10)**.

The behaviour data was subjected to Principal Component Analysis (PCA) to reveal differences between gyrodactylid behaviour in white light and red light. The behaviour parameters that showed the greatest differences between white light and red light (one-way ANOVA) were chosen (*i.e*. maximum velocity, meander and fractal dimension) and checked for normality (the remaining parameters were found to be too variable to show any patterns in behaviour). The maximum velocity data was found to be skewed, so was log transformed to normalise it. Eigen values for Factors 1 and 2 were 66.3% and 25.8%, respectively, describing a total of 92.1% of the variation in the data. The PCA plot shows two distinct groups according to behaviour in white light and red light, although some individuals in white light were grouped with those in red light (Figure 5). Examination of the individual tracks confirmed that those individuals in white light that were grouped with those in red light exhibited behaviour typical of those in red light (*i.e*. long, sinuous tracks).

### Chemical efficacy

**Figure 6:**
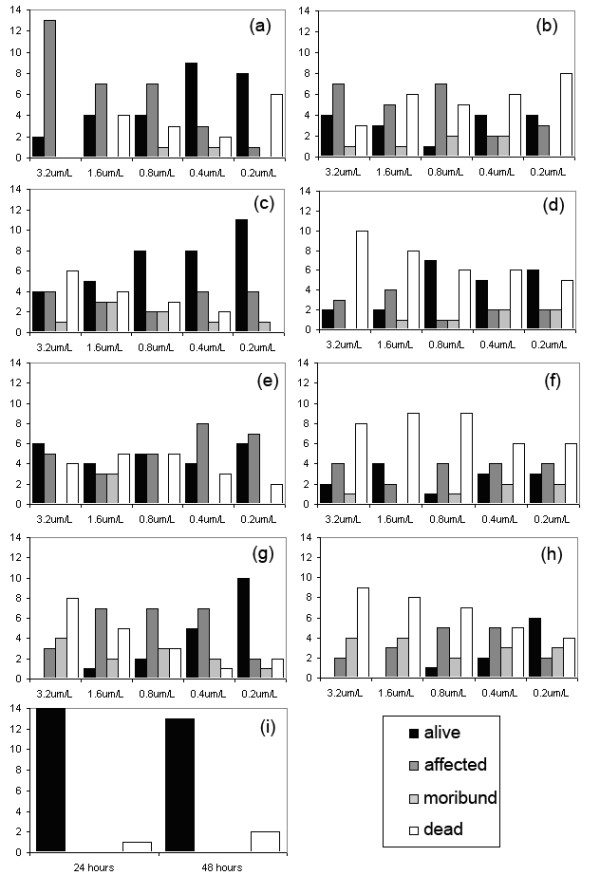
**Effect on *Gyrodactylus *of (a) chlordimeform after 24 h, (b) chlordimeform after 48 h, (c) octopamine after 24 h, (d) octopamine after 48 h, (e) clonidine after 24 h, (f) clonidine after 48 h, (g) amitraz after 24 h, (h) amitraz after 48 h and (i) control**. (y axes = number of individual gyrodactylids, n = 15 for each compound tested).

All of the four compounds affected *Gyrodactylus *and produced involuntary muscular contractions (spasms) when normal body extension was attempted. A mortality of 10% was seen in the control group after 48 hours, although no muscle spasms were observed. As the positive reference, the highest concentration of 3.2 μM of chlordimeform affected 87% of gyrodactylids after 24 h as denoted by limited movements (Figure 6a). However, after 48 h 27% of gyrodactylids were unaffected (Figure 6b) suggesting that (i) the muscular spasms may only be temporary at that concentration; (ii) the gyrodactylids needed to be at a particular physiological state before they became susceptible; (iii) the persistence of the compound affects its efficacy. As there was no clear trend in the numbers of dead, moribund and affected gyrodactylids (Figure 6a, b), it was not possible to accurately calculate EC50 values for chlordimeform.

Octopamine had a dose dependent response after 24 h, with 73% of gyrodactylids being either affected, moribund or dead at the highest concentration of 3.2 μM, compared to 27% at the lowest concentration of 0.2 μM (EC50 = 0.89 μM (0.46-1.94 μM)) (Figure 6c). After 48 h the majority (67%) of the gyrodactylids were dead at 3.2 μM (Figure 6d). Numbers of affected and moribund gyrodactylids were low for all concentrations (7%-27%) after 48 h suggesting that the optimum exposure time for octopamine is between 24 and 48 h. The 48 h EC50 for octopamine was 0.25 μM (0-0.54 μM).

Clonidine was effective after 24 h with 60% of gyrodactylids being either affected, moribund or dead at both 3.2 μM and 0.2 μM (Figure 6e). After 48 h this figure had increased to 87% at 3.2 μM and 80% at 0.2 μM (Figure 6f). As there was little difference in the number of affected gyrodactylids between the highest and lowest doses, it is possible that either the concentration range selected was too narrow to determine the effective range or there are other factors affecting the efficacy of the compound. Therefore, it was not possible to accurately calculate EC50 values for clonidine. However, as the number of affected and moribund gyrodactylids was low after 48 h (7%-27%), it is suggested that, similar to octopamine, the optimum exposure time for clonidine is between 24 and 48 h.

Amitraz was the most effective of the compounds tested with 100% of gyrodactylids being either affected, moribund or dead after 24 h at the highest concentration of 3.2 μM (53% dead) (Figure 6g). At 0.2 μM 66% remained unaffected with 20% being either affected or moribund. The 24 h EC50 for amitraz was 0.31 μM (0.18-0.44 μM). After 48 h 60% were dead at 3.2 μM and 27% were dead at 0.2 μM (Figure 6h). As there were a considerable number of gyrodactylids either affected or moribund after 48 h (33-47%), and the numbers either affected, moribund or dead after 48 h were similar to those after 24 h, it is likely that the optimum exposure time for amitraz is longer than 48 h. The 48 h EC50 value for amitraz was 0.18 μM (0.05-0.27 μM).

## Discussion

These results suggest that gyrodactylids are more active in the dark than in light and therefore imply that they possess some form of photoreceptor. Watson and Rohde [21] found sensory receptors in *Gyrodactylus *sp., which closely resemble photoreceptors found in other platyhelminths [22,23]. The light/dark experiment shows a significant difference in the distance travelled between those gyrodactylids in the dark and those exposed to light. However, as this experiment only records the start and end position of the parasite, the trial assumes that parasites have travelled in a straight line and, therefore, it is impossible to quantify their movements during the period of the experiment *i.e*. whether they follow a straight or sinuous path. This does, however, suggest that there may be differences in the distance travelled by gyrodactylids under different lighting conditions.

Although parasite tracks cannot be determined in the "dark", they can be measured under red and infrared light. By recording and tracking all the movements of individual *Gyrodactylus *it is possible to quantify their movements. While most of the measured movement parameters (velocity, distance travelled, turn rate) were higher for those gyrodactylids in red light than those in white light, none of the differences were significant. This is an indication of the wide variation in behaviours, resulting in large deviations from the mean. Conversely, meander and fractal dimensions were lower for gyrodactylids in red light than those in white light, indicating less complex tracks than those in white light. By using the movement parameters showing the greatest differences between white and red light it was possible to discriminate between the two lighting conditions using PCA. This suggests that the different conditions do result in different behaviours, although more replicates would be required to state categorically whether there are significant differences in their movements.

Observations of the tracks showed that gyrodactylids in white light often had unidirectional tracks, whereas those in red light were generally more sinuous. However, in several individuals the converse was true. Therefore, it appears that exposure to a specific cue (*e.g*. red or white light) does not always elicit a behavioural response typical of the majority of individuals exposed to the cue.

The difference in behaviour in red and white light may relate to their natural behaviour *in situ*. The long sinuous tracks of the gyrodactylids in red light, which had lower complexity and meander than those in white light, may indicate a host-seeking behaviour. Covering a large surface area as quickly as possible may allow them to identify chemical or physical cues used in host location. For example, ciliary structures likely to be photoreceptors found in *Gyrodactylus *sp. [21] may be involved in a shadow response [24], allowing gyrodactylids to detect a potential host moving overhead whilst attached to the substrate. In comparison, the behaviour exhibited by the gyrodactylids in white light (uni-directional tracks or limited movements) may indicate a response to either seek shade or conserve energy in anticipation of darkness. This implies that host-seeking behaviour is more likely to occur in dull or dark conditions. Host transmission may be more favourable at night depending on host behaviour *e.g*. if hosts are less active at night and aggregate with other hosts. Transmission during darkness may also minimise the chances of being eaten by hosts that forage during the day.

Orientation with respect to directional light requires photoreceptors with pigment shields. Although structures assumed to be light receptors have been found in gyrodactylids [21] they have no associated pigment shields. Therefore, it is likely that directional choices made by individual gyrodactylids are random and not related to directional light cues.

Host transmission may be associated with particular maturity stages of individual gyrodactylids, *e.g*. when newborn or after giving birth. However, no correlation was found between the behaviour patterns of individual *Gyrodactylus *and their maturity status. Although this does not necessarily indicate that maturity status is not linked to tranmission, (as the gyrodactylids had already been physically removed from their hosts) it does suggest that light may provide a stronger behavioural cue than maturity status, once they are detached.

The distances travelled by gyrodactylids in this study give an indication of the transmission potential via the substrate. In the tracking experiment, gyrodactylids in red light travelled a mean distance of 6.32 cm, which equates to 3.03 m over a 24 h period and in white light travelled a mean distance of 4.04 cm, equating to 1.94 m over 24 h. Transmission rates are temperature dependent and activity may increase at higher temperatures [25], indicating the dispersal and transmission potential via the substrate for detached gyrodactylids. Comparing the distances travelled in the tracking experiment with those in the experiment investigating lighting conditions, gyrodactylids travelled significantly further in the dark than in white light, suggesting that distances travelled by gyrodactylids in the dark may be even greater.

Of the four octopaminergic compounds tested, all had an effect on gyrodactylids. The initial effect was to induce muscular spasms as the parasites attempted to extend their bodies. Prolonged exposure resulted in death. It is not known if this response reflects an interaction at the peripheral or central nervous system, but does imply the presence of octopaminergic receptors. Although chlordimeform severely affected the parasites, amitraz had an even stronger effect, even at low concentrations down to 0.2 μM. Only chlordimeform at higher concentrations and amitraz significantly affected the parasites after 24 h. With octopamine and clonidine the full effect was not seen until after 48 h. This has implications for use of this type of treatment in the field, as prolonged exposure (24+ h) may be required to have any significant effect on gyrodactylids, although this delay between application and effect may be shortened with another compound due to pharmacokinetic considerations. As octopamine is a natural biogenic amine, it will be subject to metabolism and uptake by the gyrodactylids so its effect will be affected by physiological processes. This may also be the case for clonidine. As chlordimeform and amitraz are synthetic compounds, they are less likely to be affected by uptake and metabolism. In addition, it should be noted that the bioassay used in this study is relatively crude. The complex behaviours of sensory host detection followed by co-ordinated tactic motor activity involve considerable complexity and it is probable that the small behavioural effects found at very low concentrations can confer considerable efficacy for control in the field.

As the survival rates of gyrodactylids off the host are 1 day at 18°C and 4 days at 3°C for *G. salaris *[4] and 2.7 days at 15°C and 4.2 days at 4°C for *G. gasterostei *[26], this type of experiment is prone to error as a result of natural mortalities. Although mortalities in the control were only 10% it is important to bear in mind the survival rates off the host when interpreting the results. To account for control mortality an appropriate correction factor must be used, such as Abbots or Schneider-Orelli.

Before any chemical treatment against *G. salaris *can be used for entire river habitats, the toxicity of the compound to human operators and to other flora and fauna must be established. An ideal effective treatment should affect the target organism, without having adverse effects on other aquatic life. However, as the desired mode of action of any octopaminergic treatment is to interfere with the subtle behaviour of gyrodactylids, the concentrations of compound required will be considerably lower than those required to kill the parasites. As octopamine modulates virtually all physiological processes in invertebrates, but shows very little activity in vertebrates, being homologous to noradrenaline in vertebrates [7], it is likely that it will have minimal effects on vertebrates at the concentrations required to disrupt physiological processes in invertebrates. No information is available on the toxicity of octopamine in fish, although results have shown that it is non-toxic to mammals [27]. However, it is likely that the toxicity of octopamine in other aquatic invertebrates is similar to that of gyrodactylids. Although it was not possible to calculate EC50 values for chlordimeform in this study, 73% of gyrodactylids were affected or dead after 48 h at the lowest concentration of 0.2 μM (0.04 mg/L), which is considerably lower than the 96 h LC50 for rainbow trout (*Oncorhynchus mykiss *Walbaum) at 13.2 mg/L [6]. Similarly, it was not possible to calculate EC50 values for clonidine. However, at 0.8 μM (0.21 mg/L) 93% of gyrodactylids were affected or dead after 48 h. Considering that the 96 h LC50 for clonidine in ide (*Leuciscus idus *(L.)) is 87 mg/L [28], it is likely that the EC50 in gyrodactylids is significantly lower. In addition, 80% of gyrodactylids were affected by clonidine after 48 h at 0.2 μM (0.053 mg/L), which is a concentration significantly lower than the 48 h EC50 for *Daphnia *of 182 mg/L [28]. Amitraz has a 24 h EC50 of 0.29 μM (8.5 mg/L) for gyrodactylids, which is higher than the 24 h LC50 in rainbow trout of 2.7-4.0 mg/L [29]. The 48 h EC50 for amitraz in gyrodactylids is 0.16 μM (4.6 mg/L), whereas in *Daphnia magna *Straus, 1820 it has been calculated as 3.4 mg/L [30]. Although the EC50 values for amitraz are of the same magnitude as the LC50 and EC50 values for trout and *Daphnia*, it is anticipated that the concentrations required to disrupt the host seeking and attachment behaviour of gyrodactylids will be considerably lower. However, this requires further investigation.

Products containing amitraz were banned in 2010 for pesticidal uses in agriculture due to concerns of human exposure and risks to the environment [31]. Chlordimeform is banned for use as an agricultural pesticide due to concerns that it is carcinogenic to humans and is toxic to aquatic life [32]. Despite these concerns, the compounds can be used to illustrate the presence of key octopaminergic pathways in gyrodactylids.

## Conclusions

This work has made a significant step forward in the observation of gyrodactylid behaviour and is the first time that movements/activity have been studied in detail, suggesting that gyrodactylids are more active in dark than light conditions. Now that the experimental procedures have been developed to observe and record gyrodactylid movements, this system can be used for a wide variety of gyrodactylid behaviour experiments. Further work is required to confirm that gyrodactylid behaviour is affected by light conditions, specifically their behaviours in white light, red light, infrared light and dark conditions. The efficacy experiments have shown that octopaminergic receptors exist in gyrodactylids, as the octopaminergic compounds tested have an effect on gyrodactylids resulting in neuromuscular disturbance and eventually death. The next logical step is to investigate the ability of affected gyrodactylids to reattach to a fish host once they have been exposed to low doses of octopaminergic compounds and whether the effect is permanent or temporary, once they have been removed from the compounds.

These initial results observing gyrodactylid behaviour and the effect of octopaminergic compounds are promising and indicate that there might be potential use of compounds affecting octopamine receptors to control gyrodactylid infections. With the constant threat of *G. salaris *entering UK waterways and the lack of any effective treatment, other than the total eradication of all river fauna using rotenone, it is important that investment is made now to develop new chemical treatments that will specifically target *Gyrodactylus *infections.

## Competing interests

The authors declare that they have no competing interests.

## Authors' contributions

AJB made significant contributions to the conception and design of the study, constructed experimental equipment, carried out data acquisition, data analysis and interpretation, and drafted the manuscript. MIGM contributed to the design of the study and data acquisition. JEB, SI and ML contributed to the study concept and were involved in critically revising the manuscript. APS supervised the study, contributed to the conception and design of the study, interpretation of results and critically revising the manuscript. All authors read and approved the final manuscript.
